# Trajectories of women's homelessness in Canada's 3 northern territories

**DOI:** 10.3402/ijch.v74.29778

**Published:** 2015-12-23

**Authors:** Rose Schmidt, Charlotte Hrenchuk, Judie Bopp, Nancy Poole

**Affiliations:** 1 BC Centre of Excellence for Women's Health, Vancouver, BC, Canada; 2 Yukon Status of Women Council, Whitehorse, YT, Canada; 3 Four Worlds Centre for Development Learning, Cochrane, AB, Canada

**Keywords:** service improvement, homeless women, interviews, Whitehorse, Yellowknife, Iqaluit, trauma-informed, mental health, substance use

## Abstract

**Background:**

*Repairing the Holes in the Net* was a 2-year, multilevel action research project designed to inform the development of culturally appropriate and gender-specific services for northern women who are homeless or marginally housed and who face mental health and substance use concerns. The study was designed to learn about the barriers and supports experienced by homeless women in the North when accessing mental health care, shelter, housing and other services; and to inform the work of northern service providers and policy advocates in a position to implement adjustments in their praxis.

**Methods:**

This article describes the trajectories of women's service access and their ideas for service improvement from 61 qualitative, semi-structured interviews conducted with homeless women in Whitehorse, Yukon (YT), Yellowknife, Northwest Territories (NT), and Iqualit, Nunavut (NU).

**Results:**

Unresolved trauma, poverty and social exclusion, inability to find and maintain housing and ineffective services emerged as interconnected and multifaceted challenges related to women's service engagement. In the face of these challenges, women displayed significant resilience and resistance, and offered important ideas for service improvement.

**Conclusions:**

The 4 interconnected systemic challenges identified in the research, coupled with specific ideas for change cited by the resilient homeless women interviewed, offer points of entry to improve service policy and delivery. Implementing trauma-informed approaches emerged as a key example of how access to, and quality of, services could be improved for homeless women in the North.

Women are a key group in the homeless and unstable housing population in Canada (1) requiring increased attention and tailored services. In Canada's 3 territorial capitals there are upwards of 1,000 homeless women (2). Most northern homeless women do not *live rough* on the streets, and their homelessness tends to be *hidden* or *relative* in that they live in unstable or unacceptable housing (1). A critical factor in the incidence of homelessness in Canada's North is the absolute shortage of housing; rental vacancy rates are very low (1.5% in Whitehorse, 3.6% in Yellowknife and 2.7% in Iqaluit) (3). The quality of housing is also an issue; the physical environment of low-cost housing is largely substandard (2,4), and overcrowding is common (5,6).

A constellation of gendered factors, well described in *You Just Blink and It Can Happen: A Study of Women's Homelessness North of 60* (2), contribute to northern women's experiences of homelessness. Importantly, most homeless women in the North have experienced gender-based violence and trauma, which are in turn linked to mental health and substance use concerns (2). Up to 90% of the homeless women in the North are of Aboriginal descent (2,7) and the legacy of colonialism and subsequent intergenerational trauma are central factors influencing their homelessness (8,9). Migration from rural communities to the capital cities for social, economic and employment opportunities; for services such as mental health services; for better housing options; or to leave difficult or violent family relationships is common in the North (7,10,11). Once in the city, many women still face a lack of financial, social and cultural resources (11) as well as unaffordable or limited housing (7). The few emergency shelters in the North are overcrowded, understaffed and not always gender-specific (12). Emergency shelters often act as permanent housing because of limited transitional and second-stage housing (2,4). There is also a drastic shortage of mental health and addiction treatment services in the North, even in larger communities (2,7).

In the territories, many communities were not formed around a sustainable economic base and crucial shortages of formal employment opportunities exacerbate these housing- and health-related challenges (6,13). Low minimum wage, part-time seasonal service or tourism jobs without benefits and self-generated incomes related to arts and crafts are often the vocational options available (6). Many northern women must depend on Income Support, and the low levels of this support make it difficult for women to break the cycle of homelessness (14).

Over the past decade, many action plans and reports have been published in each territory on overall health and social care (e.g. 15); mental health and addictions (e.g. 16); anti-poverty strategies (e.g. 17,18); homelessness and housing (e.g. 12,19); and community programs, assets and needs (e.g. 20). These reports have identified and acknowledged cultural values, how social determinants of health operate, the importance of collaboration and guiding principles and priorities. Although housing and related needs associated with social determinants of health have been well documented, and many common recommendations have been made, there remains a need for concerted action.

## Research approach

*Repairing the Holes in the Net* was a 2-year, multilevel action research project to inform culturally appropriate and gender-specific services for homeless and marginally housed northern women. The study used a variety of methods, including conducting 61 semi-structured interviews with women to answer the research questions: (a) What is the trajectory of northern women's homelessness? (b) What are their experiences with accessing services? and (c) What are their suggestions for improving services? The interview questions are appended.

The study received ethics approval from the University of British Columbia's Office of Research Ethics, and research licenses were obtained in each territory. Women were recruited at community-based services (7 in Iqaluit, 15 in Yellowknife and 19 in Whitehorse) using posters and with assistance from service providers. In Iqaluit, all materials were provided in both English and Inuktitut. Women were eligible if they were over 18 years old, spoke English or Inuktitut (in Iqaluit) and were accessing services for mental health and housing concerns. Trained local research assistants (RAs) in each territory conducted the interviews. Interested participants were provided with a description of the project, a consent form and the plain language brochure *Your Rights in Research*, which had been adapted for this project. RAs scheduled interviews 1 week following initial contact, allowing participants time to reflect on their participation and identify any questions. Women were interviewed in a location of their choice, typically the service where they were recruited, but a few chose another location they deemed safe and confidential. Women received an honorarium, and transportation and childcare costs were covered. Interviews lasted between 15 and 90 min, with the average around 30 min.

All interviews were digitally recorded and transcribed verbatim. In Iqaluit, 7 women completed the interview in Inuktitut. These interviews were translated before being transcribed by the RA (first language is Inuktitut) who had conducted the interview. Effort was undertaken to ensure the safety and confidentiality of the participants. Women were assigned identification numbers, and no names or identifying information were collected. During the informed consent process, participants were assured they did not need answer any question they did not feel comfortable answering, and if they decided to end the interview they would still receive their honorarium. Counselling was offered to participants who wanted support after the interview.

The data was analysed using a collaborative coding process, whereby the study team met face to face for an iterative identification of themes. Ten themes were established: 1) trajectory of women’s mental health and homelessness, 2) key issues in their lives, 3) women’s strengths and goals, 4) services used, 5) entry point, 6) barriers for access, 7) strengths of services, 8) limitations of services, 9) cultural aspect of services, and 10) potential service and policy adjustments. Three interviews were collectively coded at this meeting, and 1 RA in each territory coded the remaining respective interviews in NVivo 8, using the themes established at this meeting. The team then met again to discuss what had emerged from the coding – creating thematic summaries and developing the *Vicious Cycles* model that is presented in the following sections.

## Results

Quotations are used throughout the document to illustrate the summarized themes. The territory and identification number of each participant are indicated following the quotation. Although no identifying information was directly collected, Table I described the demographic characteristics self-reported by the participants.

**Table I:** Self-reported demographic characteristics of northern homeless women interviewed, by territory

	Total	Nunavut	NWT	Yukon
	n=61	n=20	n=20	n=21
Age				
Mean (years)	38.4	33.8	33.7	42.4
Range	19–56	19–56	21–47	32–55
Not mentioned (n, %)	32 (52%)	13 (65%)	9 (45%)	10 (48%)
Race (n, %)				
Aboriginal/First Nation	22 (36%)	0 (0%)	9 (45%)	13 (62%)
Inuit	20 (33%)	18 (90%)	2 (10%)	0 (0%)
White	7 (11%)	0 (0%)	4 (20%)	3 (14%)
Not mentioned	12 (20%)	2 (10%)	5 (25%)	5 (24%)
Participants with children (n, %)				
Yes	45 (74%)	13 (65%)	17 (85%)	15 (71%)
No	9 (15%)	3 (15%)	2 (10%)	4 (19%)
Not mentioned	7 (11%)	4 (20%)	1 (5%)	2 (10%)

### Services used

Women reported using 3–14 types of services (mean=7.3), illustrating their diverse needs. Women in Whitehorse reported using the most services, a reflection of the greater number of services available to them, compared with the other territories. The upper range underestimates the actual number of services women accessed, as similar services were grouped together. Most women did not relate their service use in a linear way, and many were unclear on their entry point into services. Table II summarizes the services used by the participants. Aspects of services described as helpful included: being free, centrally located, child friendly and gender specific; having respectful and flexible workers who showed kindness and understanding; having consistent and transparent rules, drop-in hours, and access to supports for daily living (e.g. food, showers, a phone, and clothing). Given the number of services women accessed, support for navigating the systems, especially outreach, was greatly valued.

When people don't judge you, that goes a long way. Understanding, compassion […] It makes people who are looking for help feel comfortable enough that they will go back. (YT, 2)

**Table II:** Summary of services used by northern homeless women interviewed, by territory

	Total	Nunavut	NWT	Yukon
	n=61	n=20	n=20	n=21
Number of services reported				
Mean	7.3	5.6	6.6	9.6
Range	(3–15)	(3–11)	(3–15)	(4–12)
Type of service (n, %)				
Counselling for mental health or addictions concerns	46 (75%)	15 (75%)	13 (65%)	18 (86%)
Family violence shelter	44 (72%)	18 (90%)	8 (40%)	18 (86%)
Income Support	40 (66%)	7 (35%)	16 (80%)	17 (81%)
Housing Authority or other property management	39 (64%)	12 (60%)	12 (60%)	15 (71%)
Social service (e.g. Family and Child Services)	38 (62%)	14 (70%)	13 (65%)	11 (52%)
Homelessness shelter	33 (54%)	9 (45%)	12 (60%)	12 (57%)
Police/justice	29 (48%)	10 (50%)	8 (40%)	11 (52%)
Other community-based resource	28 (46%)	1 (5%)	9 (45%)	18 (86%)
Food-based service	20 (33%)	2 (10%)	3 (15%)	15 (71%)
Educational programming	17 (28%)	1 (5%)	10 (50%)	6 (29%)
Hospital	16 (26%)	4 (20%)	4 (20%)	8 (38%)
Indigenous cultural or wellness service	15 (25%)	3 (15%)	1 (5%)	11 (52%)
Primary health care/nurses station	14 (23%)	3 (15%)	7 (35%)	4 (19%)
In-patient addiction or mental health treatment (in Territory)	11 (18%)	N/A	6 (30%)	5 (24%)
Alcoholics Anonymous	11 (18%)	2 (10%)	4 (20%)	5 (24%)
In-patient addiction or mental health treatment (out of Territory)	10 (16%)	5 (25%)	1 (5%)	4 (19%)
Service for disability support	9 (15%)	1 (5%)	0 (0%)	8 (38%)
Harm reduction service	8 (13%)	N/A	N/A	8 (38%)
Alcohol or drug detox	7 (11%)	N/A	1 (5%)	6 (29%)
Church	5 (8%)	0 (0%)	3 (15%)	2 (10%)
Legal Aid	5 (8%)	3 (15%)	1 (5%)	1 (5%)
Crisis line	2 (3%)	2 (10%)	0 (0%)	0 (0%)

### Trajectories, challenges and ideas for service improvement

**Fig. 1:**
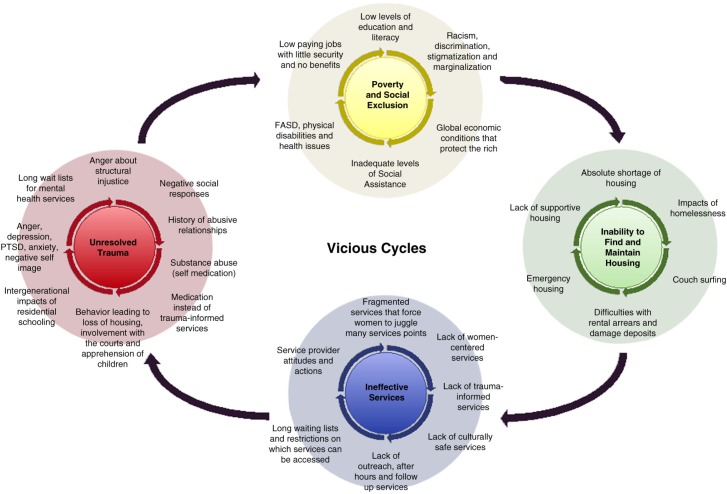
Vicious cycles contributing to northern women's homelessness.

Four overarching themes emerged from women's descriptions of the trajectory of their homelessness and their experience of accessing services: (a) unresolved trauma, (b) poverty and social exclusion, (c) inability to find and maintain housing and (d) ineffective services. Participants described how a number of *vicious cycles* made it difficult to find housing, food security and to heal emotionally; these interconnected cycles are visually depicted in Fig. 1.

### Theme 1: unresolved trauma

Traumatic experiences were at the root of many women's life challenges. Witnessing and experiencing violence was common. Most women spoke of adverse childhood experiences, including family histories of abuse, violence and addiction. Women described being introduced early to patterns of unhealthy relationships and use of substances to cope with stress, which in turn shaped their behaviour as adults.

Experiences of physical, verbal, financial, emotional and sexual abuse by current or past partners were very common. This abuse occurred many times throughout women's lives, often with multiple partners. Women described the impact of these abusive relationships, including lack of trust, fear and social withdrawal. Almost half described at least 1 incident of fleeing to seek other shelter, many returning to the abusive partner because they lacked resources, out of a sense of obligation or for the sake of their children.

He's violent too […] There's numerous times when I have to grab my baby and run out with just socks, right in the middle of the winter time. (NT, 16)

Many Aboriginal women recognized the abuse and trauma in their lives as related to colonization and residential school experiences. Women who themselves had attended residential schools described how they *blacked out* (YT, 15) or were *haunted* (NT, 19) by images of past abuse. They described their experience as one of loss – of tradition, family, language and themselves. Women described the traumatic impact of displacement, modernization and colonization in their lives, leading to the loss of traditional ways of life, increased family dysfunction and alcohol use.

Unresolved grief from losing husbands, children, siblings and other family members to tragic deaths or child apprehension was a common thread in the women's stories. Suicide, fires caused by unsuitable living conditions and accidents were also described.

[…] I was going through rough times. While I was pregnant I lost both my mother's and my father's side – their father, and my mother's boyfriend's daughter to suicide. (NU, 9)

Unresolved trauma and grief were identified as contributing to mental ill-health and problems with substance use. More than half of the women described ongoing struggles with addiction. They saw how their substance use both contributed to their daily challenges and brought relief from circumstances they felt unable to control.

It kind of takes away the pain that you're feeling, emotionally […] It helps, but it numbs the pain from your past. (NT, 14)

Many women mentioned mental health concerns, such as depression, anxiety, insomnia, anger, grief, despair, loneliness and agoraphobia. They spoke of the pain of untreated mental health issues. Many women reported a time when they had felt suicidal, and 6 women described attempting suicide.

I tried to kill myself before […] it's such a scary feeling, just thinking about it. Like, I went to that extent. […] Very, very sad, how people end their lives. When they end their lives it's because they feel alone. (NU, 3)

Some described how leaving their home communities had contributed to feelings of dislocation and poor mental health.

[…] I feel depressed, you feel sad, lonely, all kinds of emotions attached to that because of your culture shock. And you miss the food, you miss the people you left behind […] (YT, 1)

Women also spoke of the shame they felt when their actions had led to the apprehension of their children, eviction from housing or criminal charges. At the same time, many understood that this violence and anger was related to their inability to find help for their mental health and trauma-related concerns.

Women suggested a number of improvements for mental health and addiction services, including more treatment options; longer in-patient treatment; more support workers, counsellors, and therapists; and programs that focus on the positive aspects of recovery. Women also saw the need for safe and supportive services that did not require them to retell their stories to access support, and for women-specific and culturally safe services. Many cited the need for trauma-specific treatment and help with grief to address the root cause of their substance use. Local supports that allow more women to stay in their home communities instead of having to move when in crisis were also an identified need.

### Theme 2: poverty and social exclusion

Poverty was a significant stressor in women's lives. Women described having to navigate a range of bureaucratic processes and intrusive investigations to access their needs for daily living, including food, clothing and medication.

You feel like a second-class citizen simply because you're poor […] Human worth is not based on how much you make […] It's not a crime to be poor. (YT, 3)

Many of the women interviewed had not completed high school; citing family instability, giving birth at an early age, taking care of siblings and having to work to support their families as factors contributing to their low levels of education. Although a few described completing high school as adults, for many, their current unstable living situations made it challenging to undertake further education. As 1 woman said, *It's not too good to do homework with a drunk around* (NT, 17). Low levels of literacy and education contributed to feelings of marginalization, embarrassment and low self-esteem.

Women described how lack of resources, instability and discrimination affected their ability to make an adequate income. Most women relied on Income Support at levels that do not match the high cost of living in the North. Women who are able to find housing and pay rent find there is little money left to cover medical costs, transportation, furniture, food, a phone etc.

Now how can you survive, like, two hundred dollars for the family of five for the whole month? (NT, 19)

Many women spoke about feeling like a second-class citizen. Being homeless and having a mental health or substance use problem increased their feelings of marginalization. Stigma contributed to their low self-esteem and self-confidence, and some women described themselves as fearful, shy, isolated, *low* (YT, 2), and scared to speak up and voice their opinions.

Several women spoke of social isolation and lack of relational support. Racism, sexism and stigmatization were commonly described experiences. Most women lacked a significant, positive social support network. Women described being abandoned by their families and friends for a variety of reasons including leaving their home communities, being blamed for abuse and assault and for their *lifestyle* (NT, 14) (i.e. being homeless and using substances).

[My] own mother would not support me or listen to me or say I was lying […] when I was sexually abused by her immediate family, she said I was lying. She abandoned me. (NU, 3)

Income Support and social services were identified as having policies that acted as barriers. Women wanted Income Support and related services to be more empathetic, helpful and understanding of individual circumstances. Some women staying in emergency shelter wanted a daily living allowance, to support getting on their feet. Many identified needing help finding work. Women wanted more services to help them with their daily needs including public washrooms, phones, healthy food and transportation.

### Theme 3: inability to find and maintain housing

Most women expressed a desire for their own safe and stable home, and understood this as a prerequisite for dealing with other issues they faced. Yet, for most this was beyond reach.

I would phone all these numbers, every number in the paper, every day, religiously, looking for a place to rent, and I'd just cry. I'd shut right down and cry. Cause nothing […] I was doing everything I needed to do, but it just wasn't going anywhere. (YT, 14)

Most women described how long waiting lists for public housing and limited private market housing affected their ability to secure a stable place to live.

In the North it's really hard to get a place. I've been waiting for—since I was nineteen, to get a place. […] Now I'm twenty-four and I still haven't got a place.–(NT, 7)

Some described living in housing that was too cold, in disrepair, had mould or sewage problems and was not safe. The shortage of housing, particularly units for single people, makes it difficult for women to move into appropriate units when their current living situation becomes unsuitable.

Kids grew up, they wanted to go live with their dad […] what was the point keeping a three-bedroom unit any more? I couldn't even keep up with the power bill, so they evicted me. (NT, 19)

All of the women interviewed described at least one experience with unstable or inadequate shelter. Most had stayed with a friend or family member because they had nowhere else to stay, and many described having to move around because they had outstayed their welcome. Women described being vulnerable to physical and sexual abuse, finding that men who allow them to *couch surf* (YT, 4) often expect sexual relations in return for shelter.

[…] we're staying in this room, bedroom, and then we had to put a blanket in the closet and she [her daughter] slept on the closet floor. And that really hurts. –NT, 13

Other women described times when they took shelter in tents, and how unsuitable this solution was particularly in the winter.

I actually live in the campground […] it's hard to sleep only five hour because it's way too cold. (YT, 7)

Although emergency shelters exist in each territory, women described inadequate shelter services and supportive housing options. Women described policies and conditions that made staying at the emergency shelters undesirable: In Yellowknife, the women’s shelter does not allow older children, women in Iqaluit’s shelter are unable to collect any income and Whitehorse’s Salvation Army shelters both men and women, limiting safety and privacy for women. The lack of supportive housing and second-stage housing contributes to women being trapped in cycles of homelessness.

Rental arrears and damage deposits can trap women in homelessness. Women cited a number of reasons for getting into a situation of arrears, including difficulty maintaining housing and paying for bills, sometimes because they or their partners spent money on alcohol or gambling; difficulties when they returned to school; insufficient levels of Income Support; and damage caused by themselves, partners, or their children. Once a woman is evicted and loses her damage deposit, she is not only responsible for paying arrears but also not eligible for a second damage deposit from Income Support.

It also put me in debt because damage deposits or the first-and-last are not covered by social assistance. (YT, 3)

Women described the significant impact that being homeless had on their life trajectories. It was difficult for many to maintain stability in their own and their children's lives, and some had sent their children to live with relatives or had them apprehended.

[…] housing breaks a lot of people's lives. (YT, 10)

Women identified the need for transitional housing with supports for women leaving treatment for addiction and mental illness; permanent second-stage housing; increased stock of good-quality, low-income housing, including units for single women, an easily accessible fund for rent and damage deposits, rent geared to income and more rent supplements. A suggestion was made that the Landlord & Tenant Act could be modified to prevent winter evictions, and housing workers and landlords could receive training on conflict resolution skills to prevent evictions that can begin a cycle of homelessness. They also suggested that housing policies should take into consideration the needs, realities and dynamics of women fleeing violence.

### Theme 4: ineffective services

Women described a complex, uncoordinated system of services that was difficult to navigate with their current resources.

[…] Like they are not very specific on how to—any of these things work. It's like it's a total different world. (NT, 7)

Narrow mandates, long waiting lists, slow follow-up times and lack of outreach services make it difficult for women to successfully navigate services, and in some cases can put their safety at jeopardy. For example, 2 women described how they were unable to take housing units when they became available because they did not receive their Income Support payments in time. A lack of family-centred services leads to situations where women need to separate from their children to stay in a shelter or attend addiction treatment in the South.

I was trying to go into a treatment program and I was basically told that my kids would have to go into child care, which is with Child Welfare. (YT, 1)

Women wanted counselling that acknowledged the role of their past trauma in their current mental health challenges. They saw how medication can mask suffering, instead of support healing and change.

I am on medication […] because I can't handle a lot of stuff out here so they've gotta [sic] drug me up so I can accept a lot of this. (YT, 4)

Women felt that the quality of services they received was often dependent on the compassion of individual staff members with whom they built positive relationships, relationships that were difficult to rely on in situations of high staff turnover. Service provider attitudes that stigmatize and punish rather than support, and the lack of capacity to respond to individual needs rather than simply follow policies were identified as barriers to effective services.

You feel belittled, right? And they make it difficult for you and they make you feel like shit for being there. They make you feel like you're taking their money. (YT, 9)

Women spoke of difficulties meeting the conditions expected of them to obtain services. Women described having to go to counselling to be reunited with their children, to do *productive choices* (NT, 18), such as education, to receive Income Support and *collect points* (NU, 11) while on waiting lists for housing. Women said when they failed to meet these conditions, they were not being resistant or non-compliant but rather these expectations were more than they could achieve with their current resources.

Women thought services would better meet their needs if they had extended hours on evenings and weekends, street-level outreach and were more visible and connected, making it easier for them to locate what they needed. They also suggested aftercare programs for women after treatment are urgently needed to break the cyclical use of services. Women wanted more culturally centred programs, such as women's circles for sharing and support, land-based healing options, programs with elders, culture-specific activities (e.g. smudging) and more involvement in defining culturally safe programming.

## Discussion

Tightly interwoven systemic and personal issues reinforce women's homelessness. The 4 vicious cycles described in our results are mutually reinforcing and illustrative of the difficulties northern women face trying to break the cycle of homelessness. Life is difficult for the women who participated in the study. At the same time, they spoke eloquently about their aspirations for the future, the strategies they use to meet daily needs and the ways they work to maintain dignity and a sense of agency in the face of societal discrimination, and seemingly insurmountable odds. Their resilience shone through expressing gratitude for service providers who go beyond the call of duty, swallowing pride to ask for help, demonstrating remarkable creativity about meeting daily living needs, avoiding and managing dangerous street situations and finding allies in the service system. Although women described their struggles, they also spoke of their strengths that, if supported, could help them achieve their aspirations for employment, improving education, healing from trauma, securing housing, participating in activities with friends and contributing to positive change in the system to benefit others. This picture of resilient women calls into question a common stereotype – that homeless women are to blame for their situation, “being the result of substance use, mental health problems, bad choices, laziness, or simple bad luck” (21, p. 7).

Our work contributes to knowledge about northern women's homelessness by exploring women's experiences of accessing services. Although the literature on northern women's homelessness is scarce, the existing literature complements our findings. The only comprehensive study of women's homelessness in the North acknowledges the cyclical nature of homelessness and mental health challenges, and the contribution of unresolved trauma to these patterns (2). Recent literature on southern homeless women also notes the impact of cycles of poverty, stress, loss of personal relationships and systemic subjection of women's experiences of homelessness (22–25). This literature also highlights the importance of histories of abuse and intimate partner violence in women's pathways to homelessness and as a barrier to seeking services (23,25).

The literature also supports what the women in our study called for: integrated, trauma-informed and women-centred services; and addressing of the social and economic factors that affect homelessness and mental health. Qualitative research with homeless women in Toronto, Ontario reveals that women value non-judgmental support, and that service provision must feel both safe and confidential (21). Trauma-informed approaches to services delivery do not require the disclosure of trauma (26,27), instead they create safe, compassionate and welcoming services that focus on not retraumatizing (28,29). Research supports that trauma-informed services are well received by consumers, lead to better outcomes and promote system change (29,30). Faced with the reality of scarce funding, and limited available housing, practicing in a trauma-informed way may prove to be central to repairing the holes in the net of existing services in the North.

The findings of this study may not be generalizable to all homeless women in the North. Our sample included women who were engaging with services at the time of recruitment, and women accessing services and choosing to participate may differ significantly from those who do not. We relied on self-reports to determine which services the women had used, and their accounts may not have been complete. Also, we limited participation to women over 19 years old who spoke English or Inuktitut, and the experiences of younger women, or those who do not speak these languages may differ from our sample.

Future research is needed to investigate how systems of care are/might more adequately provide empowering, trauma-informed, integrated (housing, Income Support, violence, mental health, substance use) support to women, to respond to the challenges posed by women in this study and thereby “repair the holes in the net.”

## Conclusions

Women experience a range of interconnected barriers making breaking the cycle of homelessness almost insurmountable, necessitating a range of shifts in policy and services. Provision of housing, including transitional and supportive housing, is essential for addressing homelessness in Canada's North. However, the findings of this study call upon us to fundamentally rethink service interventions to shift from stigma to respect for women's resiliency and goals; to a strength-based approach that focuses on restoring holistic wellness rather than merely addressing symptoms; to service delivery from a trauma-informed stance, so that basic safety, cultural safety and empowerment can be realized; and for providing a visible and accessible net of linked services that address the clearly identified shelter, Income Support, food security, health, vocational, and educational needs of women and their families.

The following are the interview questions for service users:

1. Can you tell me a bit about what service(s) you are accessing these days? The services can be about helping you with housing challenges, mental health issues or any related issues (such as violence or substance use services) that you see as connected to your wellbeing.
  - What influenced you to choose these particular services over others you might have used?
  - What is it that made these services attractive or useful to you?
2. We are interested in services you have used in the past as well as the one(s) you are using today. What was your experience with these services?
  - What was helpful and what was not so helpful?
    [A tool was provided to map the services that the woman had used by age if that was helpful.]
    Prompt: Encourage the woman to discuss the experience she had with each service she marks in or names. Why did she choose it? Was it a good or “not so good” experience?
3. As you look over this map [or list] of the services you have used, what are the qualities of the services that have been important or helpful to you?
  - On the contrary, what are the qualities of the services that were not helpful or made you uncomfortable?
4. Are there types of services that are not available that you would like to have access to?
  - If so, can you describe what services, or qualities of services, you wish were in place?
5. Do you have any other comments about accessing services and how they have affected you?
